# Transduodenal surgical ampullectomy for intra-ampullary papillary tubular neoplasm (IAPN): A case report

**DOI:** 10.1016/j.ijscr.2021.106253

**Published:** 2021-08-01

**Authors:** Susan Pradhan, Krishna Mohan Adhikari, Romi Dahal, Sumita Pradhan, Ramesh Singh Bhandari

**Affiliations:** Department of GI and General Surgery, Tribhuvan University Teaching Hospital, Institute of Kathmandu, Nepal

**Keywords:** Ampulla, Ampullectomy, Case report, Intra-ampullary papillary tubular neoplasm, IAPN

## Abstract

**Introduction and importance:**

Intra-ampullary papillary tubular neoplasms (IAPNs) are relatively rare kind of neoplasms occurring in the region of the papilla which exhibit significant malignant transformation. The patient was concerned about his pain and the possibility of malignancy.

**Case presentation:**

We report a case of a 47-year-old male who presented with persistent upper abdomen pain. Following detail investigations, he was diagnosed as IAPN and managed by transduonal ampullectomy (TDA).

**Clinical discussion:**

The insidious onset of IAPN along with its high risk of malignancy makes it mandatory for its proper treatment. Although, endoscopic approach is advantageous for initial therapy, it has some technical difficulties. Hence TDA forms the cornerstone in the management of IAPN with good prognosis.

**Conclusion:**

Transduodenal ampullectomy is a safe and feasible option for IAPN. It can be the first choice of treatment in selected cases where endoscopic papillectomy is not available.

## Highlights

• Intra-ampullary papillary tubular neoplasms (IAPNs) are unusual neoplasms capable of potential malignant progression.
• We report a rare case of IAPN which was treated through transduonal ampullectomy (TDA).
• TDA can be adopted as an alternative treatment approach to manage IAPNs with great efficacy and fair long-term results.

## 1 Introduction

Intra-ampullary papillary tubular neoplasms (IAPNs) are rare tumoral intraepithelial neoplasia occurring within the ampulla comprising 0.5% of all gastrointestinal tumors [1]. IAPNs are highly analogous to pancreatic or biliary intraductal papillary and tubular neoplasms as evidenced by their papillary and/or tubular growth, variable cell lineage, and spectrum of dysplastic change. Owing to their malignant potency, it is important to early recognize and treat IAPNs [1], [2]. We report an adult with IAPN who was treated by transduodenal ampullectomy (TDA) in a tertiary level university hospital of Nepal. This case report has been reported in line with the scare guidelines [3].

## 2 Presentation of case

A 47-year-old male presented to our Surgical Gastroenterology Out-patient clinic with pain in upper abdomen for 9 months which was insidious in onset, gradually progressive, dull aching, mild to moderate in severity and non-radiating. It was not associated with fever, vomiting, abdominal distension, jaundice, melena, loss of appetite or weight loss. There was no history of patient receiving any medication in the past. There was no any significant family history.

**Fig. 1:**
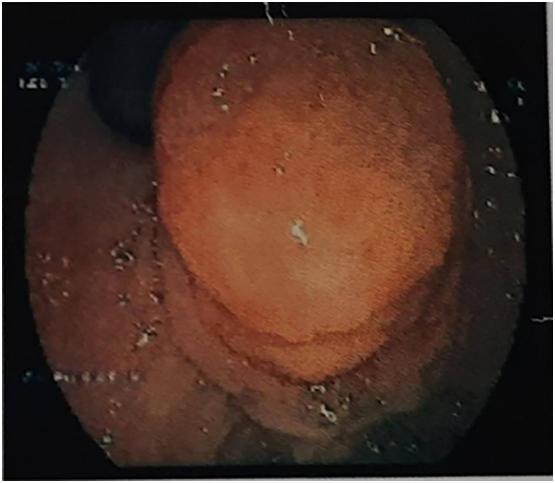
UGI Endoscopic showing bulky ampulla with growth arising from the ampulla of Vater.

**Fig. 2:**
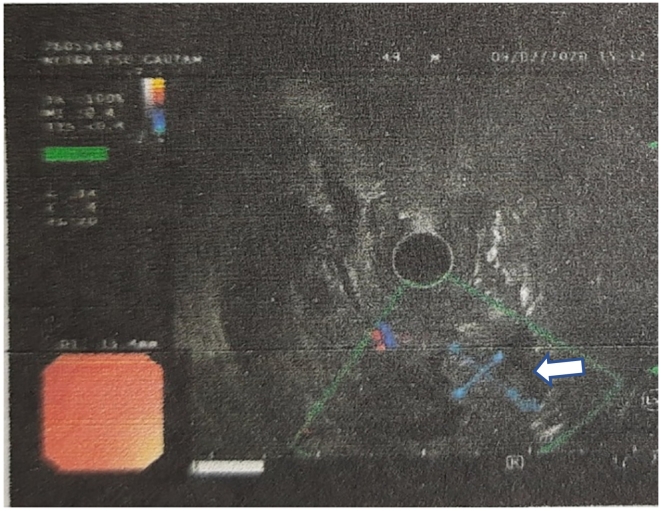
EUS showing hyperechoic lesion at ampulla.

**Fig. 3:**
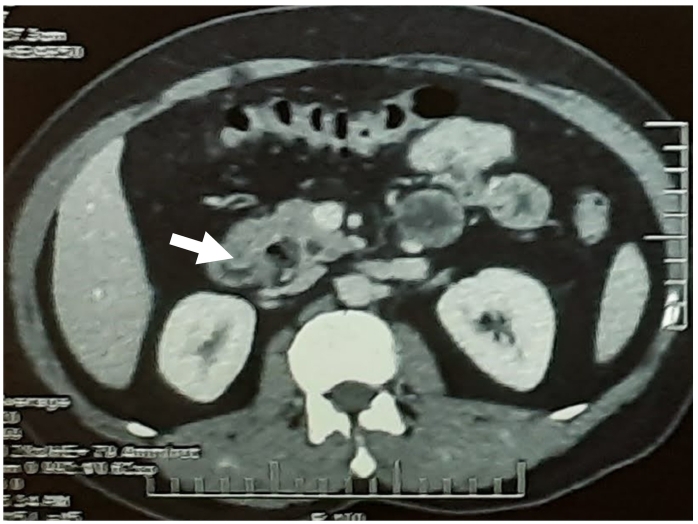
CECT abdomen and pelvis showed a well-defined homogenously enhancing lesion in region of ampulla with features of biliary obstruction.

For these complains the patient underwent upper gastrointestinal (UGI) endoscopy which showed bulky ampulla with growth arising from the ampulla of vater (Fig. 1). Endoscopic biopsy was taken and the histopathologic examination (HPE) of mass showed features suggestive of adenoma. Later on, he underwent Endoscopic Ultrasonography (EUS) for better characterization of the mass, which showed hyperechoic lesion at ampulla (Fig. 2) with the HPE suggestive of low grade dysplasia. A computed tomography (CT) scan of his abdomen and pelvis was done which showed a well-defined homogenously enhancing lesion in region of ampulla with features of biliary obstruction (Fig. 3).

All hematological and biochemical findings were normal except total bilirubin, 4 mg/dL (normal 0.3 to 1.5); direct bilirubin, 1.0 mg/dL (normal 0.01 to 0.35) and alkaline phosphatase,262 IU/L (normal 30 to 140). The tumor markers assessed were cancer antigen 19.9(CA 19.9), 16.6 U/mL (normal 0 to 37) and carcinoembryonic antigen (CEA), 1.69 U/mL (normal 0 to 30).

He was planned for TDA. The procedure was done by a senior consultant HPB surgeon. Upper midline incision was given and exploration of the abdominal cavity and the liver was done. The duodenum and pancreatic head were mobilized from the retroperitoneum by a Kocher maneuver. Palpation of the pancreatic head was done to exclude an underlying pancreatic head tumor. A longitudinal duodenotomy, the ampulla of Vater was identified. Cannulation of the ampulla was done. Local excision of the ampulla was performed by electro-cautery with careful identification of the pancreatic and bile ducts. Both ducts were cannulated to prepare for reconstruction, a separate opening of the pancreatic and bile ducts was created by single 5–0 suture (duct-to-mucosa) and two-layer closure of the duodenal wall completed reconstruction.

**Fig. 4:**
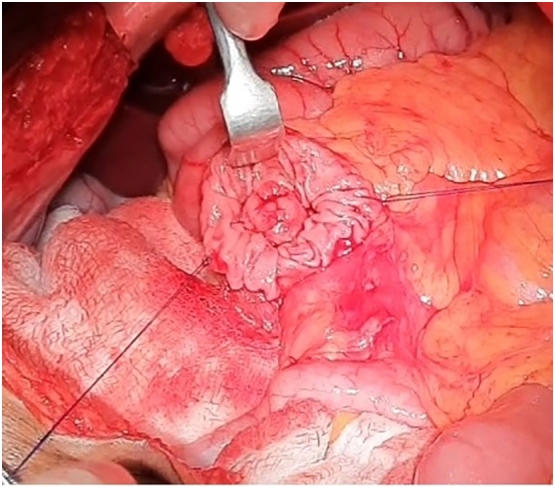
Transduodenal approach showing the bulge at the ampulla of approximately 3 × 1 cm.

**Fig. 5:**
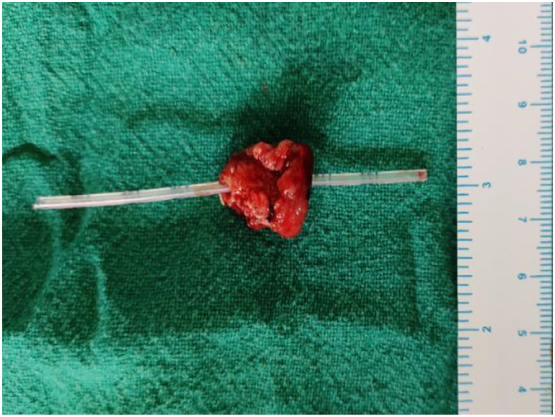
Specimen of the ampulla (measuring 3 × 1.5 cm) with cannula through the duct.

Intraoperative findings; liver and peritoneum were normal. There was no ascites. There was a mass of size 3 × 1.5 cm in ampulla (Fig. 4, Fig. 5).

**Fig. 6:**
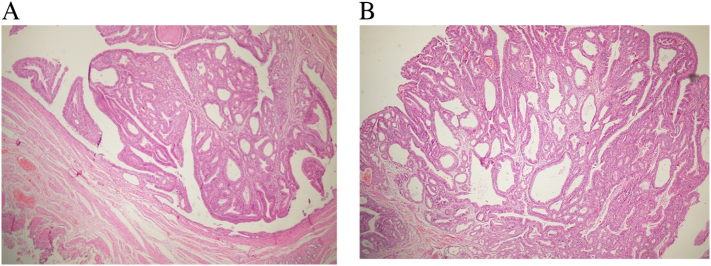
(A) and (B) Histopathology slide showing polypoidal tumor composed of closely packed tubular glands lined by pseudostratified columnar epithelium with loss of polarity and moderate nuclear atypia of intra-ampullary papillary tubular neoplasm of high grade and stalk was free of tumor.

HPE reports showed polypoidal tumor composed of closely packed tubular glands lined by pseudostratified columnar epithelium with loss of polarity and moderate nuclear atypia suggestive of IAPN of high grade and stalk was free of tumor (Fig. 6). His postoperative days were uneventful and was discharged on 6th postoperative day. His last follow up was at 6th month and he was doing well. The patient was satisfied with the treatment received.

## 3 Discussion

Intra-ampullary papillary tubular neoplasms (IAPNs) are rare neoplasms characterized by exophytic growth, variable cellular lineage, and a spectrum of dysplasia. More than 80% of the cases show a mixture of low and high-grade dysplastic foci within the same lesion. IAPNs constitute 33% of primary ampullary tumors and 5.5% pancreaticoduodenectomy/ampullectomies with neoplasms [1]. Macroscopically, most IAPNs have minimal exophytic growth at the duodenal surface aspect of the papilla of Vater. However, sometimes they form a mucosal protuberance in the duodenal lumen due to the underlying proliferative process, which fills the intra-ampullary/ductal lumen. The duodenal orifice of the ampulla is often wide, irregular, and occasionally ulcerated. On cut sections of ampulla, IAPNs are characterized by polypoid or papillary (granular-exophytic) lesions, which fill the ampullary channel (or the intra-ampullary tips of both the CBD and pancreatic duct). Microscopically, there is a variable degree of papillary and tubular configuration. About a one fourth of the lesions are predominantly composed of papillary elements, another one fourth are tubular; however, more than half of the cases exhibit a substantial mixture of the two growth patterns i.e. tubulopapillary [4].

Clinical presentation of patients with IAPNs is unspecific and mostly related to obstruction of the common bile duct or pancreatic duct. Typical symptoms are abdominal pain, jaundice, weight loss, diarrhea, diabetes, nausea and vomiting. Age peak is from the sixth to seventh decade [1]. Laboratory findings can be normal or show signs of cholestasis with or without cholangitis. The diagnosis is usually done with UGI endoscopy, ERCP or EUS guided biopsy. However, even if the biopsy is negative, it cannot reliably exclude the possibility of malignancy. CECT abdomen is helpful to stage the disease. The prognosis for IAPNs (both noninvasive and invasive cases considered) is significantly better than that of other invasive carcinomas of the ampulla and pancreatic ductal adenocarcinoma [2], [5].

In most cases of IAPN without infiltration or metastases, surgical resection is recommended [6]. Poley et al. promote three surgical options: Endoscopic ampullectomy or TDA or Pancreaticoduodenectomy (PD). Indications for surgical resection are an adenoma greater than 2 cm in diameter, evidence of lymph node involvement, and evidence of ingrowth of adenoma into the bile or pancreatic duct [7]. Endoscopic ampullectomy is globally recognized as a first-choice procedure for benign ampullary pathologies. The advantages of endoscopic ampullectomy are low morbidity and mortality rates compared with surgical procedure [8], [9], [10]. However, endoscopic ampullectomy was technically difficult in our setting due to the limited experience. PD is associated with a relatively high surgical morbidity (25–50%) and mortality (approximately 5%). TDA is a less invasive and simple technique, which could potentially provide equivalent clinical outcomes for early ampullary tumors compared to radical PD. [11]

Kim et al. had studied 31 patients with ampulla of Vater tumors retrospectively. He investigated the safety and availability of TDA and endoscopic papillectomy (EP) were compared. There was no significant difference in the occurrence of complications between the TDA group and EP group (*p* = 0.145), and the resection margins were negative in both groups. This study suggests that TDA is as safe as EP for treating benign periampullary tumors [7]. TDA is an underestimated surgical procedure, which can be performed safely with high long-term efficacy. It can be implemented in clinical algorithms for patients with benign pathologies of the ampulla of Vater [8]. Although EP is the first choice of IAPN, it is not readily available due to lack of technically skilled manpower. Therefore, TDA can be first choice in IAPN in resource limited settings where endoscopic resections cannot be done.

## 4 Conclusion

IAPN is one of the rare benign ampullary pathologies. Endoscopic treatment represents an important tool that requires expertise. Transduodenal ampullectomy is a safe and feasible option for IAPN. It can be the first choice of treatment in selected cases where endoscopic papillectomy is not available.

## Funding

None.

## Ethical approval

Nothing to declare.

## Consent

Written informed consent was obtained from the patient for publication of this case report and accompanying images. A copy of the written consent is available for review by the Editor-in-Chief of this journal on request.

## Authors' contribution

Ramesh Singh Bhandari (RBS), Sumita Pradhan (SP) and Romi Dahal (RD) = Study concept, Data collection, and surgical therapy for the patient.

Susan Pradhan (SP*), Romi Dahal (RD) and Krishna Mohan Adhikari(KMA) = Writing - original draft preparation.

SP*, RD and KMA = Editing and writing.

RSB and SP = Senior author and manuscript reviewer.

All the authors read and approved the final manuscript.

## Registration of research studies

Not applicable.

## Guarantor

Professor Ramesh Singh Bhandari. Department of GI and General Surgery, Tribhuvan University Teaching Hospital, Institute of Kathmandu, Nepal, Phone: +977-9841270203 P.O. Box: 1522.

## Provenance and peer review

Not commissioned, externally peer-reviewed.

## Declaration of competing interest

None.
